# Effectiveness of a Web-Based Guided Self-help Intervention for Outpatients With a Depressive Disorder: Short-term Results From a Randomized Controlled Trial

**DOI:** 10.2196/jmir.4861

**Published:** 2016-03-31

**Authors:** Robin Maria Francisca Kenter, Pim Cuijpers, Aartjan Beekman, Annemieke van Straten

**Affiliations:** ^1^Faculty of Behavioural and Movement SciencesDepartment of Clinical, Neuro and Developmental PsychologyVrije Universiteit AmsterdamAmsterdamNetherlands; ^2^EMGO Institute for Health Care and ResearchVU University Medical CentreAmsterdamNetherlands

**Keywords:** depression, outpatient clinics, Internet-based treatment, problem solving therapy, specialized mental health care

## Abstract

**Background:**

Research has convincingly demonstrated that symptoms of depression can be reduced through guided Internet-based interventions. However, most of those studies recruited people form the general population. There is insufficient evidence for the effectiveness when delivered in routine clinical practice in outpatient clinics.

**Objective:**

The objective of this randomized controlled trial was to study patients with a depressive disorder (as defined by the Diagnostic and Statistical Manual of Disorders, fourth edition), as assessed by trained interviewers with the Composite International Diagnostic Interview, who registered for treatment at an outpatient mental health clinic. We aimed to examine the effectiveness of guided Internet-based self-help before starting face-to-face treatment.

**Methods:**

We recruited 269 outpatients, aged between 18 and 79 years, from outpatient clinics and randomly allocated them to Internet-based problem solving therapy (n=136), with weekly student support, or to a control condition, who remained on the waitlist with a self-help booklet (control group; n=133). Participants in both conditions were allowed to take up face-to-face treatment at the outpatient clinics afterward. We measured the primary outcome, depressive symptoms, by Center for Epidemiological Studies Depression scale (CES-D). Secondary outcome measures were the Hospital Anxiety and Depression Scale Anxiety subscale (HADS-A), Insomnia Severity Index questionnaire (ISI), and EuroQol visual analog scale (EQ-5D VAS). All outcomes were assessed by telephone at posttest (8 weeks after baseline).

**Results:**

Posttest measures were completed by 184 (68.4%) participants. We found a moderate to large within-group effect size for both the intervention (d=0.75) and the control (d=0.69) group. However, the between-group effect size was very small (d=0.07), and regression analysis on posttreatment CES-D scores revealed no significant differences between the groups (b=1.134, 95% CI –2.495 to 4.763). The per-protocol analysis (≥4 sessions completed) results were also not significant (b=1.154, 95% CI –1.978 to 7.637). Between-group differences were small and not significant for all secondary outcomes. Adherence to the intervention was low. Only 36% (49/136) received an adequate dosage of the intervention (≥4 of 5 sessions). The overall treatment satisfaction was moderate.

**Conclusions:**

Internet-based problem solving therapy is not more effective in reducing symptoms of depression than receiving an unguided self-help book during the waitlist period at outpatient mental health clinics. The effect sizes are much smaller than those found in earlier research in the general population, and the low rates of adherence indicate that the acceptability of the intervention at this stage of treatment for depressed outpatients is low. However, taking into account that there is much evidence for the efficacy of Internet-based treatments, it is too early to draw firm conclusions about the effectiveness of these treatments in outpatient clinics as a whole.

**Trial Registration:**

Netherlands Trial Register NTR2824; http://www.trialregister.nl/trialreg/admin/rctview.asp?TC=2824 (Archived by WebCite at http://www.webcitation/ 6g3WEuiqH)

## Introduction

Depressive disorders are highly prevalent [1]. They affect 16% of the population on a lifetime basis [2,3] and are expected to become the leading cause of disability in high-income countries by 2030 [4]. Depression substantially impairs patients’ daily life and reduces quality of life to a great extent. It is therefore essential to reduce the burden of depression as much as we can. A reduction in the associated health care uptake and improved work productivity is also beneficial to society as a whole.

Given the high prevalence and demand for mental health care, it is important for mental health care providers to optimize the use of their scarce resources. Long waiting lists before the start of psychological treatment are undesirable, but not uncommon. Patients who are referred to specialized mental health care rarely receive immediate access to psychological treatments [5]. In the Netherlands, for example, the time between registration and the first treatment session is normally at least 6 weeks. This time might be used more efficiently by deploying Internet-based self-help treatments [6], which might make treatment more effective and efficient if patients require fewer or no face-to-face sessions afterward.

Previous controlled studies have demonstrated that guided self-help is clinically effective in diverse populations [7-9] and that it has effects comparable with those of face-to-face treatments [10,11]. However, most studies on Internet treatments have been conducted with self-referred participants recruited from the community through advertisements. Those patients probably would not have received any treatment if these trials had not been conducted. This population might be different from those who seek help at outpatient mental health clinics, who might have more severe symptoms and different expectations, as they expect to receive regular face-to-face treatment. This might influence the way they view and respond to Internet treatment. The current evidence base on the effectiveness of guided self-help delivered by the Internet to clinical populations who are referred to outpatient mental health clinics is small. Although some research exists on the effectiveness of Internet treatments in routine psychiatric care [12], most research has focused on self-referred participants from the general population [13,14] or in primary care settings [15].

Internet-based guided self-help interventions are not offered as a first step for patients who are waiting for outpatient mental health clinics, and no trials have specifically evaluated whether starting with Internet-based treatments is effective in those settings. It is important to examine whether the effects found in previous trials can be replicated in clinical populations in outpatient care, as guided self-help has many advantages that could be of great benefit. The advantages include improved access to treatments for patients, less waiting time, and potential costs savings, as these treatments require significantly less therapist time than do conventional treatments and put less strain on therapeutic resources. Introducing Internet-delivered self-help interventions as a start of treatment might bridge the treatment gap and can possibly speed up clinical recovery [16].

### Aims and Hypotheses

We conducted this randomized controlled trial among patients with a depressive disorder (as defined by the *Diagnostic and Statistical Manual of Disorders*, fourth edition, DSM-IV) who registered for help in an outpatient mental health clinic. The aim of the study was to examine the effects of guided Internet-based self-help before starting face-to-face therapy. We expected the intervention group to report significant improvements in symptoms of depression at posttest, relative to the control group.

## Methods

### Ethics Statement and Trial Registration

The trial was approved by the Medical Ethics Committee of the VU Medical Centre Amsterdam (registration number 2011.223) and has been registered with the Netherlands Trial Register (NTR2824). We collected written informed consent from all participants.

### Design and Sample Size

Our study was a randomized clinical trial examining the effects of an Internet-based problem solving therapy with scheduled email guidance before starting face-to-face therapy in outpatient clinics. The control group stayed on the waiting list and received a self-help book but without any form of guidance. The reason for sending this self-help book was to motivate people to participate in the trial.

The full study design can be found elsewhere [17]. We made changes to the protocol by not using analysis of covariance for data analysis, but instead using regression analysis. Moreover, we managed to recruit 269 patients instead of the 248 participants as outlined in the protocol.

### Recruitment

We recruited patients directly after registration for regular face-to-face treatments at the participating outpatient clinics. From December 2011 to August 2013. a total of 828 patients consented to share their contact details with the research team. We screened patients presenting with symptoms of a mood disorder by telephone using the Composite International Diagnostic Interview for presence of a major depressive disorder. Subsequently, we checked other inclusion and exclusion criteria. Baseline measures were administered by phone to eligible patients (N=269). After each inclusion an independent researcher allocated patients to either the intervention group (n=136) or the control group (n=133) using a random allocation sequence stratified by location in blocks of 6, 8, and 10 generated by the independent researcher in the program Random Allocation Software version 2.0 (Informer Technologies, Inc).

### Inclusion and Exclusion Criteria

To be eligible for this study, participants had to (1) be aged ≥18 years, (2) be waiting for face-to-face treatment at the participating clinics, (3) fulfill the DSM-IV [18] criteria for major depressive disorder as a primary diagnosis, (4) have access to the Internet, and (5) have adequate proficiency in Dutch. Comorbidity other than bipolar or psychotic disorders was allowed. We excluded patients presenting with suicidal ideation from the trial. We also excluded patients who started antidepressant medication, switched type, or changed dosage 12 weeks before or during the first phase of the trial. Patients who were ineligible remained on the waiting list for face-to-face treatment.

We temporarily excluded new participants from any outpatient clinic where the waiting time fell below 8 weeks, until that clinic’s waiting time for new patients again exceeded 8 weeks. We made this decision so as not to measure the effects of active treatment by the clinic.

### Intervention

We based the Internet intervention on problem solving therapy using self-examination therapy as a general framework [19]. The intervention’s intent is to teach skills that help patients to regain control over their problems. Patients learn to determine what really matters to them (session 1) and learn structured strategies to solve those problems that are related to what matters (sessions 2, 3, and 4). Furthermore, attention is paid to thinking less negatively about the unimportant problems (session 3) and to learn to accept those situations that cannot be changed (session 4). As the intervention had already proven to be effective in community samples in different studies [13,14,20,21], we presented the intervention to the patients as a way to make a head start in their treatment during the waiting time.

The intervention has been described in more detail in several other studies [13,14,17]. In brief, it is a short, structured, and highly manualized generic intervention consisting of 5 weekly sessions. Each session contains structured homework assignments on which the participants receive weekly online feedback by a coach. The total amount of time coaches had for responding to each patient’s assignment was about 15 to 20 minutes. The feedback was of a nontherapeutic nature and was aimed at helping participants to become familiar with the presented techniques. In the first session, participants were required to determine what is important in their lives. Next to this, they had to make a list of current problems and worries and divide them into 3 categories: (1) not important (ie, not related to the list of important things), (2) important and unsolvable (eg, permanent loss of health or a loved one), or (3) important and solvable. In the second, third, and fourth sessions, participants were offered various coping skills related to each of the categories of problems. The main focus was on adopting a structured 6-step approach when encountering important, solvable problems. This structured approach consisted of identifying the problem; finding solutions; selecting one solution; creating a plan to solve the problem with this solution; executing the plan; and evaluating the plan. The last week of the intervention was reserved for both the reflection on long-term goals and the development of a structure to achieve these goals.

Participants could only move on to the next session once they had submitted the exercise in the previous session and when the research team had released feedback on this session. When they did not finish a session, participants received an email from their coach to encourage them to carry on and to offer assistance in case there were specific problems preventing further use of the intervention.

During the trial, we migrated the website to an updated version to safeguard participants’ data according to Dutch law and to fix defects in website functionality. The content of the platform remained unchanged.

### Control Condition

To increase participation rates in the control group, this group received a self-help book format without any form of guidance. The book was sent as is, without further instructions. Previous research showed that self-guided interventions resulted in only small effect sizes on levels of depression [7-9].

Participants in both conditions already had an appointment for face-to-face treatment at the clinic scheduled after the waitlist period. Waiting times were not affected by participation in this study.

### Assessments

All outcome measures were administered by phone by trained research assistants at baseline and posttest (8 weeks from randomization). This short interval was to ensure that patients had not started with conventional psychotherapy at the clinics.

### Primary Outcome Measure

The primary outcome, symptoms of depression, was measured by the Center for Epidemiological Studies Depression scale (CES-D) [22], consisting of 20 items with total scores ranging between 0 and 60. Higher scores indicate higher levels of depression and a score of ≥16 indicates a clinical level of depression. This questionnaire has been tested in various populations and has been found both valid and reliable [23].

### Secondary Outcome Measures

Secondary outcome measures were symptoms of anxiety, symptoms of insomnia, quality of life, and mastery. We used the Anxiety subscale of the Hospital Anxiety and Depression Scale (HADS-A) to measure symptoms of anxiety. The Anxiety subscale consists of 7 items, with scores ranging from 0 to 21; higher scores indicate higher levels of anxiety. The HADS has been shown to be reliable in Dutch populations [24].

We administered the Insomnia Severity Index questionnaire (ISI) [25] to measure both the concerns associated with the perceived level of insomnia, and symptoms and consequences of insomnia. Each item is rated on a scale of 0 to 4; a higher score indicates more severe insomnia. ISI has been found to be internally consistent and reliable [26].

We used the EuroQol visual analog scale (EQ-5D VAS) [27] to measure the patients’ self-rated health. Endpoints (0, 100) are labelled “Best imaginable health state” and “Worst imaginable health state.”

We measured the amount of perceived control in a person’s life by the Pearlin Mastery Scale [28]. The scale consists of 7 distinct items that are rated on a 4-point scale. Higher scores indicate more perceived control; scores range from 7 to 35. The scale has good reliability.

### Process Outcome Measures

Process outcome measures were adherence, general satisfaction with the treatment, and the *Alles Onder Controle* (Everything Under Control) satisfaction questionnaire.

We defined treatment adherence (0=low adherence, 1=adherence) as completing at least 4 of the 5 lessons within the given time frame of 5 weeks. Participants who completed at least 80% of the Web-based material would have been exposed to the majority of the interventions described in the manual. We defined lesson completion as (1) reading the exercises, (2) doing the exercises, (3) receiving feedback on exercises, and (4) reading the feedback. Participants could move to the next session only when they had completed the exercises and opened the feedback from the coach, which we marked as read. We monitored these data through the intervention platform.

Satisfaction with the Internet intervention was measured by the Client Satisfaction Questionnaire-8 (CSQ-8), which consists of 8 questions; each question is scored on a Likert-type scale from 1 to 4 [29]. The questionnaire addresses several elements that contribute to overall service satisfaction and is reported in a single dimension of overall satisfaction. A high internal consistency has been reported [29].

The *Alles Onder Controle* satisfaction questionnaire was designed specifically to investigate to what degree participants were satisfied with this Internet-based intervention. It includes questions on the number of sessions completed and, if applicable, the reasons for not finishing the course. The questionnaire further researches satisfaction with the separate elements of the intervention, such as the quality of the feedback, the clarity of the website, and the appropriateness of the examples.

### Statistical Analysis

We examined differences between groups on baseline characteristics by performing chi-square tests for categorical variables and 1-way analysis of variance for continuous variables. Baseline data were available for all participants. We analyzed missing values on the outcome measures in agreement with the intention-to-treat (ITT) principle, as per the CONSORT statement [30]. We used a logistic regression analysis with backward stepwise method to explore whether participants’ characteristics could predict missing data at posttest.

Missing endpoints at posttest (31.6%, 85/269) were imputed using the multiple imputation function in IBM SPSS Statistics 20 (IBM Corporation), yielding 30 imputations with 30 iterations using the multiple imputation option with predictive mean matching. Predictors for the imputing procedure were pretest and nonmissing posttest scores, age, education level, sex, and randomization status.

We calculated the intraclass correlation to examine nonindependence of observations at the outpatient clinic level. We did not deem a multilevel approach for analyzing data to be necessary. Therefore, we preformed linear regression analyses to examine posttreatment differences between the intervention and the control group. The baseline score of the dependent variable was added as a covariate to adjust the outcome for baseline differences. Differences between the intervention and the control condition were denoted by a regression coefficient (B) and 95% CIs.

We also expressed the magnitude of the effects as Cohen *d* effect sizes by dividing the difference in mean scores of the 2 groups by their pooled standard deviation (Xexp-Xctrl/SDpooled). Effect sizes <0.2 are considered to be small, of 0.5 are moderate, and of 0.8 are large [31]. We calculated the effect sizes for all participants (ITT). We performed all analyses on both completer-only data and the imputed file. Furthermore, we performed a per-protocol analysis based on treatment completers (completed ≥4 sessions of the intervention).

## Results

### Sample Characteristics

**Figure 1:**
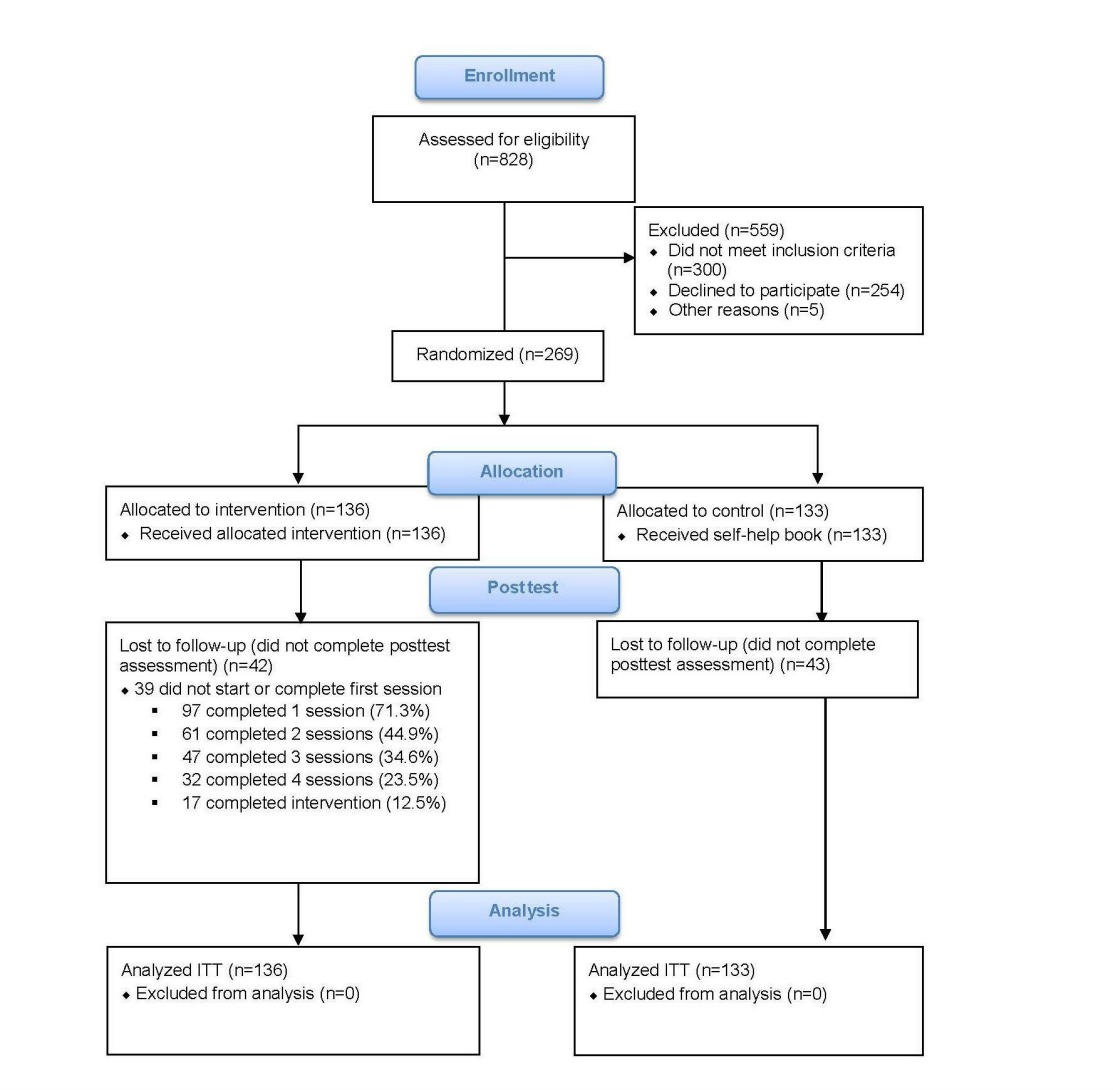
Participant flowchart.

Figure 1 shows the flow of participants through the trial. A total of 828 patients who signed up at the outpatient clinics agreed to be contacted by the research team. Of these, 300 did not meet the inclusion criteria, 254 declined to participate or did not provide informed consent, and 5 had other reasons for not participating in the study. We randomly allocated the remaining 269 participants to either the intervention group (n=136) or the control group (n=133).

Table 1 presents baseline data. The mean age of participants was 38.0 (SD 11.4), and 145 (53.9%) were women. Most of the participants were born in the Netherlands (223/269, 82.9%) and had an education level from middle (105/269, 39.0%; upper secondary general education, secondary vocational education, postsecondary education) to high (111/269, 41.3%; specialized vocational education, university or college education). Their income was mostly below the Dutch average (171/269, 63.6%) based on the average income for the years 2012/2013, which was €33.000. There were no significant differences between intervention and control group on demographic variables or any of the outcome measures at baseline.

**Table 1:** Baseline characteristics of outpatients receiving Internet-based therapy (intervention) or placed on a waitlist (control) for treatment of depression.

Participant characteristics		Intervention (n=136)	Control (n=133)	Total (N=269)	*P* value^a^
**Sex, n (%)**					.25
	Male	58 (42.6)	66 (49.6)	124 (46.1)	
	Female	78 (57.4)	67 (50.4)	145 (53.9)	
**Age (years)**					
	Mean (SD)	38.6 (10.5)	37.4 (12.3)	38.0 (11.4)	.41
	Range	18–64	18–79	18–79	
Born in the Netherlands, n (%)		111 (81.6)	112 (84.2)	223 (82.9)	.46
Income less than average^b^, n (%)		86 (63.2)	85 (63.9)	171 (63.6)	.94
**Educational level** ^c^ **, n (%)**					.69
	Low	29 (21.8)	24 (17.6)	53 (19.7)	
	Middle	51 (38.3)	54 (39.7)	105 (39.0)	
	High	53 (39.8)	58 (42.6)	111 (41.3)	
Antidepressant medication, n (%)		29 (21.3)	38 (28.6)	67 (24.9)	.17
**Treatment history, n (%)**					
	Psychological treatment	80 (58.8)	77 (57.9)	157 (58.4)	.88
	Internet treatment	3 (2.2)	7 (5.3)	10 (3.7)	.19
	Self-help book	31 (22.8)	29 (21.8)	60 (22.3)	.85
	None	22 (16.2)	20 (15.0)	42 (15.6)	.87
**Symptom measures, mean (SD)**					
	Depression (CES-D^d^)	37.0 (11.6)	35.2 (12.2)	36.1 (11.9)	.22
	Anxiety (HADS-A^e^)	12.4 (3.9)	12.6 (4.7)	12.5 (4.3)	.67
	Insomnia (ISI^f^)	13.9 (6.5)	13.7 (6.1)	13.8 (6.3)	.39
	Quality of life (EQ-5D VAS^g^)	53.1 (17.4)	50.3 (17.1)	51.7 (17.3)	.20
	Mastery (Pearlin Mastery Scale)	20.1 (4.0)	19.5 (3.5)	19.8 (3.8)	.20

^a^Tested with *t* test for continuous variables or chi-square test for categorical variables.

^b^Average income for 2012/2013 = €33.000 (Central Bureau for Statistics).

^c^Low: no education, preprimary, primary, lower secondary education, compulsory education, initial vocational education. Middle: upper secondary general education, secondary vocational education, postsecondary education. High: specialized vocational education, university or college education.

^d^CES-D: Center for Epidemiological Studies Depression scale.

^e^HADS-A: Hospital Anxiety and Depression Scale, Anxiety subscale.

^f^ISI: Insomnia Severity Index questionnaire.

^g^EQ-5D VAS: EuroQol visual analog scale.

### Study Attrition

Of the 269 patients who started pretest assessment, 184 (68.4%) completed the posttest assessment. We tested whether there were significant differences in baseline characteristics, as described in Table 1, between study completers and noncompleters. Noncompleters were more likely to be female, younger, and less educated and had a lower income. There were no differences in any of the clinical characteristics such as depression severity. Table 2 shows the statistically significant differences between noncompleters and completers; not reported baseline characteristics were not significant.

### Treatment Adherence

Adherence to the intervention was low. About a third (49/136, 36%) received an adequate dosage of the intervention. They completed either 4 (n=32; 23.5%) or 5 (n=17; 12.5%) lessons. Treatment expectancy and credibility measures at baseline predicted higher adherence (B=1.086, *P=*.019). Not all participants reported reasons for nonadherence (85/119); however, the reported reasons for dropout where mostly lack of energy (35/85), that the course was not a priority (20/85), technical problems (16/85), no benefits from the course (12/85), or feeling better (2/85).

### Effects

Both groups showed significant improvements between baseline and posttest on all outcomes measured (see Table 3).

**Table 2:** Baseline differences between outpatients with depressive disorder who completed and did not complete posttest assessments.

Characteristics	Completers (n=184)	Noncompleters (n=85)	*P* value^a^
Female, n (%)	90 (48.9)	55 (64.7)	.02
Age in years, mean (SD)	39.2 (11.5)	35.4 (11.0)	.01
Higher education^b^, n (%)	86 (46.7)	25 (29.4)	.02
Low income^c^, n (%)	106 (57.6)	65 (76.5)	.049

^a^Tested with chi-square or *t* test.

^b^Specialized vocational education, university or college education, doctorate, postdoctorate, and equivalent degrees.

^c^Less than average income in the Netherlands (average €33.000 for 2012/2013).

**Table 3:** Results of outcome measures for outpatients receiving Internet-based therapy (intervention) or placed on a waitlist (control) for treatment of depression: completers and intention-to-treat (ITT) sample.

Measure		Completers sample		ITT sample		Statistic ITT sample		Effect size^c^	
		Pretest mean (SD)	Posttest mean (SD)^a^	Pretest Mean (SD)	Posttest mean (SD)^a^			Within group	Between group
						B^b^	95% CI		
**CES-D** ^d^						1.13	–2.495 to 4.763		0.07
	Control	35.2 (12.2)	27.5 (12.3)	35.2 (12.1)	25.9 (14.9)			0.69	
	Intervention	37.0 (11.6)	25.9 (12.6)	37.0(11.6)	27.0 (15.1)			0.75	
**HADS-A** ^e^						0.47	–.793 to 1.722		0.09
	Control	12.6 (4.7)	10.5 (4.2)	12.6 (4.6)	10.0 (5.5)			0.52	
	Intervention	12.4 (3.9)	10.0 (4.3)	12.4 (3.9)	10.5 (5.4)			0.41	
**ISI** ^f^						0.27	–1.424 to 1.955		0.04
	Control	13.7 (6.1)	11.5 (5.4)	13.7 (6.1)	11.3 (7.6)			0.35	
	Intervention	13.9 (6.5)	11.2 (5.6)	13.9 (6.4)	11.6 (7.2)			0.34	
**EQ-5D VAS** ^g^						–1.76	–7.162 to 3.648		0.07
	Control	50.3 (17.1)	57.3 (19.0)	50.3 (17.1)	59.3 (23.1)			0.44	
	Intervention	53.1(17.3)	59.4 (19.3)	53.1 (17.3)	57.6 (23.3)			0.22	
**Pearlin Mastery Scale**						–.027	–.968 to .419		0.10
	Control	19.5 (3.5)	27.1 (2.6)	19.5(3.5)	27.5 (3.1)			2.42	
	Intervention	20.1 (4.0)	27.4 (2.6)	20.1 (4.0)	27.2 (2.9)			2.03	
**CSQ-8** ^h^						0.49	–.907 to 1.886		0.05
	Control	NA^i^	20.5 (4.6)	NA	20.1 (5.3)			NA	
	Intervention	NA	20.1 (4.8)	NA	20.4 (6.1)			NA	

^a^Controlled for baseline scores.

^b^Regression coefficient.

^c^Cohen *d*.

^d^CES-D: Center for Epidemiological Studies Depression scale.

^e^HADS-A: Hospital Anxiety and Depression Scale, Anxiety subscale.

^f^ISI: Insomnia Severity Index questionnaire.

^g^EQ-5D VAS: EuroQol visual analog scale.

^h^CSQ-8: Client Satisfaction Questionnaire-8.

^i^NA: not administered at that time point.

### Primary Outcome Measure

The analysis showed that both groups improved significantly in depression scores from pre- to posttest (control group: B=0.56, 95% CI 0.34–0.78, *P*<.001; intervention group: B=0.61, 95% CI 0.38–0.84, *P*<.001). The within-group effect size Cohen *d* was moderate to large for both the intervention (*d*=0.75) and the control (*d*=0.69) groups. However, the between-group effect size was very small (*d*=0.07). Regression analysis on posttreatment CES-D scores controlling for pretreatment scores found no significant differences in posttreatment scores between the intervention group and the control group (*B*=1.134, 95% CI –2.495 to 4.763).

The per-protocol analysis (≥4 sessions completed) also did not yield significant results on the posttest differences in depressive symptoms between the 2 groups (B=1.154, 95% CI –1.978 to 7.637).

### Secondary Outcome Measures

The within-group effect sizes were large for mastery, moderate for anxiety, and small to moderate for insomnia and quality of life. Between-group differences were small for all secondary outcomes, and differences between the groups were not significant.

### Treatment Satisfaction

Treatment group participants who completed the posttreatment satisfaction questionnaire (n=92) were moderately positive about the intervention (average grade 7 out of 10, SD 1.08). A total of 69 reported that the intervention was useful to them and 29/92 reported that the program was either very useful or mostly helpful. A minority (7/92) rated the program as not helpful to their problems and needs.

Participants were mostly satisfied with the quality of the feedback, and only 4/92 were not satisfied with the feedback from their coach. Most participants in the intervention group (53/92) reported that they would have preferred to start their treatment with a face-to-face session with a therapist. Another 24 stated that they would have preferred to start with the Internet intervention and the remaining 15 participants reported that they didn’t prefer one treatment modality over the other. The estimated mean score of the CSQ-8 was 20.4 (SD 6.1) among the 32 participants indicating a moderate degree of satisfaction.

### Adverse Events

During the trial, 1 control group participant committed suicide. The participant had started face-to-face treatment at the outpatient clinic.

## Discussion

### Principal Findings

Although the efficacy of guided Internet-based treatment for depression has been firmly established [32], there is insufficient evidence of its effectiveness when delivered in routine outpatient mental health services. This study examined the effectiveness of a guided Internet-based self-help intervention, based on problem solving therapy, for patients with a depressive disorder who were awaiting face-to-face treatment at outpatient mental health clinics. We expected the guided Internet-based intervention to lead to better outcomes for patients compared with our control condition. The results did not support our hypothesis. Both groups demonstrated significant improvements in depression from pre- to posttest, with moderate to large within-group effect sizes. However, the between-group effect sizes were small and nonsignificant. The guided Internet-based intervention was not more effective than being on the waitlist with an unguided self-help book.

Compared with previous studies that examined the same Internet-based intervention against a pure waitlist control condition in community samples, the between-group effect size of our study is much smaller (*d*=0.47 [13], *d*=0.50 [14], current trial: *d*=0.07), but the change in depressive symptoms in our intervention group is similar to the change in symptoms in previous trials (ΔCES-D=11.3 [13], ΔCES-D=9.0 [14], current trial: ΔCES-D=10.0). The main difference is that our control group, against our expectations, showed significant improvements from pre- to posttest. This improvement in symptoms might have been caused by the self-help book, although previous trials found only small effects on depressive symptoms [7-9]. Other explanations might be that the reductions in symptoms in the control group were caused by test procedures (eg, telephone administered questionnaires), by the use of antidepressant medication (intervention group: 21.3%, control group: 28.6%; see Table 1) or by spontaneous improvement, which is not uncommon in depression. In any case, the reduction in symptoms in the control group may have led to a more conservative estimate of the between-group effect sizes.

### Comparison With Prior Work

Most previous trials examined the effects of Internet treatment in community samples [13,14], general practitioners’ offices [15], or specialized Internet clinics [12]. The results of these trials are much better in terms of adherence and effectiveness than the results of our trial involving regular outpatient mental health clinics. One explanation for the difference in outcome might be that this Internet intervention, based on problem solving therapy, wasn’t effective for depressed patients who signed up for face-to-face treatment at regular outpatient clinics. This argument can be supported with the finding of low adherence to the intervention.

Another explanation might be the differences in study design. Previous trials [13,14] tested the exact same intervention against a waitlist control group that received no self-help book or any other treatment but were assigned to a pure waitlist condition. The previous trials also tested the intervention in community samples where participants actively signed up for Internet treatment without face-to-face sessions, as opposed to our participants, who signed up for regular treatment at specialized mental health clinics.

Furthermore, the initial levels of depression were higher in our sample than in the previous trials (baseline CES-D=29.9 [14], 31.9 [13], current trial: 37.0). The low-intensity Internet-based intervention may not have been suitable for severely depressed patients in this stage of their treatment. Perhaps these patients would have benefited more from an intervention of higher intensity. The low adherence might indicate that this short self-help intervention does not meet the needs of severely depressed patients, despite the findings from a recent meta-analysis of individual patient data [33] that showed that patients with more severe depression at baseline derived at least as much clinical benefit from low-intensity interventions as less severely depressed patients.

The results of our study also compare poorly with those of other studies of Internet-based cognitive behavioral therapy (iCBT) in routine care [12,15,34]. The difference in effectiveness might be explained by how the intervention was offered and the intensity of the treatment. In the Swedish iCBT clinic, for example, a face-to-face diagnostic assessment was offered before the start of Web-based treatment, a licensed psychologist carried out the treatment, and the iCBT consisted of more treatment sessions than we had in our intervention. Furthermore, those participants signed up for iCBT as their first choice of treatment, whereas in our study patients signed up for face-to-face treatment and were afterward referred to Internet treatment that was carried out by coaches of the university (masters-level students). The coaches’ feedback in our study was not of a therapeutic nature, but was highly templated and focused on guiding the participant through the intervention. Even though the participants were mostly satisfied with the quality of the feedback, it did not lead to high adherence. Perhaps more intensive and therapeutic feedback from a clinician of the outpatient clinic would increase the completion rates. Also, the pace of 1 lesson per week may have been too rapid, and giving extra time could have led to higher completion rates. Furthermore, the patients in our study were already scheduled for a face-to-face appointment at the clinics, regardless of whether they finished the intervention. This might have lowered the motivation to persist with Internet treatment.

A final remark is that we accepted patients who had previously received treatment, including psychological treatments, for participation in our study. About 60% reported that they received psychological treatment before participating in this trial, 4% had received Internet treatment, and 22% had already used a self-help book; 16% reported no treatment history (see Table 1). Although those who had not received any prior psychological treatment completed more Internet sessions, this was not significant. The inclusion of participants who had previously received psychological treatment might have affected treatment outcome. Compared with studies that did not allow inclusion of participants who received prior psychological treatment, the participants in our sample might have been more treatment resistant than first-time treatment seekers. However, this remains speculative.

Overall, the results compare poorly with the results from previous studies in community samples and routine care. The low adherence to this intervention is a cause for concern because it is far lower than generally found in Internet-based interventions for depression [12,34-36].

### Limitations

Some limitations of this randomized controlled trial are important to acknowledge. The first important limitation, as mentioned above, was the poor adherence to the intervention. Although we see adherence as an indicator of acceptability of the treatment, it may also be related to treatment outcome. The low adherence rates limited the extent to which our participants were exposed to the content of the intervention, thereby lowering the possible effects of the intervention.

Second, offering a self-help book without guidance may have influenced the between-group results, as the waitlist group may have improved using the self-help book. When we designed this trial, the literature showed small effect sizes for unguided self-help (ranging from *d=*0.06 to *d=*0.28 [7-9]) and larger effect sizes for guided Internet interventions (ranging from *d=*0.61 to *d=*0.78 [7,8]) compared with waitlist controls. However, more recent studies that have directly compared guided with newer self-guided Internet-based interventions found no difference in clinical outcomes for depression and anxiety [37,38]. Moreover, 28 (21%) of the participants in the control group stated that they had read the entire self-help book and 17 (12.8%) read half or more of the self-help book. These numbers indicate that the control group had read as much of the material as the intervention group did.

Third, not all participants completed posttest measurements. We corrected for missing values by using multiple imputation. However, imputing 31% of the data may have led to unreliable estimates. Regardless of the method for handling missing values, data on treatment satisfaction were available for only 92 of the intervention groups participants, of whom only 32 completed 4 or more lessons. The majority of the intervention group was thus not exposed to a large part of the intervention, and these results should be interpreted with caution.

In spite of these limitations, our study extends the current literature in an important way. To date, no randomized controlled trials have been conducted within existing regular outpatient mental health clinics that examined the effectiveness of Internet-based problem solving therapy for depression. In this trial we examined actual patients at actual outpatient clinics and were quite successful with the recruitment as compared with trial recruitment in primary care, which can be more difficult [39]. One study of the effects of iCBT for depression recruited only 7 participants from 11 general practices in 8 months [40]. In our trial, however, we were able to randomly allocate just over 50% of the 528 eligible patient. In all, 254 declined to participate in the study (before being screened), and 300 (36.2%) did not meet inclusion criteria. In total we were able to include 32.5% (269/828) of all referrals.

### Conclusion

This study showed that Internet-based problem solving therapy is not more effective in reducing symptoms of depression than unguided self-help during the wait time at outpatient clinics. The between-group effect sizes were much smaller than those found in earlier trials in the general population and in primary care. Together with the low rates of adherence, the results indicate that the acceptability of the intervention at this stage of treatment in outpatient clinics is low for patients with depression. However, taking into account that there is much evidence for the efficacy of Internet-based treatments in the research setting, in particular for iCBT, it is too early to draw firm conclusions about the effectiveness of these treatments in outpatient clinics as a whole. While several countries are increasingly offering Internet treatments in clinical practice, more research is needed that examines who benefits from Internet-based treatments in outpatient settings. Only then can we draw firm conclusions about the feasibility, acceptability, and effectiveness of Internet-based interventions for a wider group of patients in regular mental health care.

## Multimedia Appendix 1

Alles onder Controle Intervention.

## Abbreviations

CES-D: Center for Epidemiological Studies Depression scale
CSQ-8: Client Satisfaction Questionnaire-8
DSM-IV: Diagnostic and Statistical Manual of Disorders, fourth edition
EQ-5D VAS: EuroQol visual analog scale
HADS-A: Hospital Anxiety and Depression Scale Anxiety subscale
iCBT: Internet-based cognitive behavioral therapy
ISI: Insomnia Severity Index questionnaire
ITT: intention to treat

## References

[1] Ayuso-Mateos JL, Vázquez-Barquero JL, Dowrick C, Lehtinen V, Dalgard OS, Casey P, Wilkinson C, Lasa L, Page H, Dunn G, Wilkinson G (2001). Depressive disorders in Europe: prevalence figures from the ODIN study. Br J Psychiatry.

[2] Kessler RC, Chiu WT, Demler O, Merikangas KR, Walters EE (2005). Prevalence, severity, and comorbidity of 12-month DSM-IV disorders in the National Comorbidity Survey Replication. Arch Gen Psychiatry.

[3] Kessler RC, Angermeyer M, Anthony JC, Demyttenaere K, Gasquet I, Gluzman S, Gureje O, Haro JM, Kawakami N, Karam A, Levinson D, Posada-Villa J, Stein DJ, Aguilar-Gaxiola S, Alonso J, Lee S, Heeringa S, Pennell B, Berglund P, Gruber MJ, Petukhova M, Chatterji S, Ustün TB, Medina Mora Maria Elena, Oakley Browne Mark A, Adley Tsang Cheuk Him (2007). Lifetime prevalence and age-of-onset distributions of mental disorders in the World Health Organization's World Mental Health Survey Initiative. World Psychiatry.

[4] Mathers CD, Loncar D (2006). Projections of global mortality and burden of disease from 2002 to 2030. PLoS Med.

[5] Kessler RC, Berglund PA, Bruce ML, Koch JR, Laska EM, Leaf PJ, Manderscheid RW, Rosenheck RA, Walters EE, Wang PS (2001). The prevalence and correlates of untreated serious mental illness. Health Serv Res.

[6] Andrews G, Cuijpers P, Craske MG, McEvoy P, Titov N (2010). Computer therapy for the anxiety and depressive disorders is effective, acceptable and practical health care: a meta-analysis. PLoS One.

[7] Richards D, Richardson T (2012). Computer-based psychological treatments for depression: a systematic review and meta-analysis. Clin Psychol Rev.

[8] Andersson G, Cuijpers P (2009). Internet-based and other computerized psychological treatments for adult depression: a meta-analysis. Cogn Behav Ther.

[9] Donker Tara, Johansson Robert, Mohr David C, van Straten Annemieke, Andersson Gerhard, Cuijpers (2011). Self-guided psychological treatment for depressive symptoms: a meta-analysis. PLoS One.

[10] Andersson G, Cuijpers P, Carlbring P, Riper H, Hedman E (2014). Guided Internet-based vs. face-to-face cognitive behavior therapy for psychiatric and somatic disorders: a systematic review and meta-analysis. World Psychiatry.

[11] Cuijpers P, Donker T, van SA, Li J, Andersson G (2010). Is guided self-help as effective as face-to-face psychotherapy for depression and anxiety disorders? A systematic review and meta-analysis of comparative outcome studies. Psychol Med.

[12] Hedman E, Ljótsson B, Kaldo V, Hesser H, El AS, Kraepelien M, Andersson E, Rück C, Svanborg C, Andersson G, Lindefors N (2014). Effectiveness of Internet-based cognitive behaviour therapy for depression in routine psychiatric care. J Affect Disord.

[13] Warmerdam L, van SA, Twisk J, Riper H, Cuijpers P (2008). Internet-based treatment for adults with depressive symptoms: randomized controlled trial. J Med Internet Res.

[14] van Straten S, Cuijpers P, Smits N (2008). Effectiveness of a web-based self-help intervention for symptoms of depression, anxiety, and stress: randomized controlled trial. J Med Internet Res.

[15] Høifødt RS, Lillevoll KR, Griffiths KM, Wilsgaard T, Eisemann M, Waterloo K, Kolstrup N (2013). The clinical effectiveness of web-based cognitive behavioral therapy with face-to-face therapist support for depressed primary care patients: randomized controlled trial. J Med Internet Res.

[16] Kenter R, Warmerdam L, Brouwer-Dudokdewit C, Cuijpers P, van SA (2013). Guided online treatment in routine mental health care: an observational study on uptake, drop-out and effects. BMC Psychiatry.

[17] Kenter RMF, van SA, Hobbel SH, Smit F, Bosmans J, Beekman A, Cuijpers P (2013). Effectiveness and cost effectiveness of guided online treatment for patients with major depressive disorder on a waiting list for psychotherapy: study protocol of a randomized controlled trial. Trials.

[18] American Psychiatric Association (2000). Diagnostic and Statistical Manual of Mental Disorders, 4th edition, text revision (DSM-IV-TR).

[19] Bowman D, Knapp S, Jackson TL, VandeCreek L (1995). Self-examination therapy: treatment for anxiety and depression. Innovations in Clinical Practice: A Source Book.

[20] Bowman D, Scogin F, Lyrene B (1995). The efficacy of self-examination therapy and cognitive bibliotherapy in the treatment of mild to moderate depression. Psychother Res.

[21] Mynors-Wallis LM, Gath DH, Day A, Baker F (2000). Randomised controlled trial of problem solving treatment, antidepressant medication, and combined treatment for major depression in primary care. BMJ.

[22] Radloff LS (1977). The CES-D scale: aself report depression scale for research in the general population. Appl Psychol Meas.

[23] Beekman AT, Deeg DJ, Van LJ, Braam AW, Van TW, De Vries M Z (1997). Criterion validity of the Center for Epidemiologic Studies Depression scale (CES-D): results from a community-based sample of older subjects in The Netherlands. Psychol Med.

[24] Spinhoven P, Ormel J, Sloekers P P, Kempen G I, Speckens A E, Van Hemert A M (1997). A validation study of the Hospital Anxiety and Depression Scale (HADS) in different groups of Dutch subjects. Psychol Med.

[25] Morin CM (1993). Insomnia: Psychological Assessment and Management.

[26] Bastien CH, Morin CM, Ouellet M, Blais FC, Bouchard S (2004). Cognitive-behavioral therapy for insomnia: comparison of individual therapy, group therapy, and telephone consultations. J Consult Clin Psychol.

[27] EuroQol Group (1990). EuroQol: a new facility for the measurement of health-related quality of life. Health Policy.

[28] Pearlin LI, Schooler C (1978). The structure of coping. J Health Soc Behav.

[29] de Brey H (1983). A cross-national validation of the client satisfaction questionnaire: the Dutch experience. Eval Program Plann.

[30] Schulz KF, Altman DG, Moher D (2010). CONSORT 2010 statement: updated guidelines for reporting parallel group randomized trials. Ann Intern Med.

[31] Cohen J (1998). Statistical Power Analysis for the Behavioral Sciences.

[32] Hedman E, Ljótsson B, Lindefors N (2012). Cognitive behavior therapy via the Internet: a systematic review of applications, clinical efficacy and cost-effectiveness. Expert Rev Pharmacoecon Outcomes Res.

[33] Bower P, Kontopantelis E, Sutton A, Kendrick T, Richards DA, Gilbody S, Knowles S, Cuijpers P, Andersson G, Christensen H, Meyer B, Huibers M, Smit F, van Straten A, Warmerdam L, Barkham M, Bilich L, Lovell K, Liu ET (2013). Influence of initial severity of depression on effectiveness of low intensity interventions: meta-analysis of individual patient data. BMJ.

[34] Ruwaard J, Lange A, Schrieken B, Dolan CV, Emmelkamp P (2012). The effectiveness of online cognitive behavioral treatment in routine clinical practice. PLoS One.

[35] Perini S, Titov N, Andrews G (2009). Clinician-assisted Internet-based treatment is effective for depression: randomized controlled trial. Aust N Z J Psychiatry.

[36] van Ballegooijen W, Cuijpers P, van SA, Karyotaki E, Andersson G, Smit JH, Riper H (2014). Adherence to Internet-based and face-to-face cognitive behavioural therapy for depression: a meta-analysis. PLoS One.

[37] Berger T, Hämmerli K, Gubser N, Andersson G, Caspar F (2011). Internet-based treatment of depression: a randomized controlled trial comparing guided with unguided self-help. Cogn Behav Ther.

[38] Farrer L, Christensen H, Griffiths KM, Mackinnon A (2011). Internet-based CBT for depression with and without telephone tracking in a national helpline: randomised controlled trial. PLoS One.

[39] Bower P, Wilson S, Mathers N (2007). Short report: how often do UK primary care trials face recruitment delays?. Fam Pract.

[40] Woodford J, Farrand P, Bessant M, Williams C (2011). Recruitment into a guided internet based CBT (iCBT) intervention for depression: lesson learnt from the failure of a prevalence recruitment strategy. Contemp Clin Trials.

