# Supplementary figures and images for: Effectiveness of a Web-Based Guided Self-help Intervention for Outpatients With a Depressive Disorder: Short-term Results From a Randomized Controlled Trial
Source: J Med Internet Res. 2016 Mar 31;18(3):e80. doi: 10.2196/jmir.4861 (PMC4832120; doi:10.2196/jmir.4861)

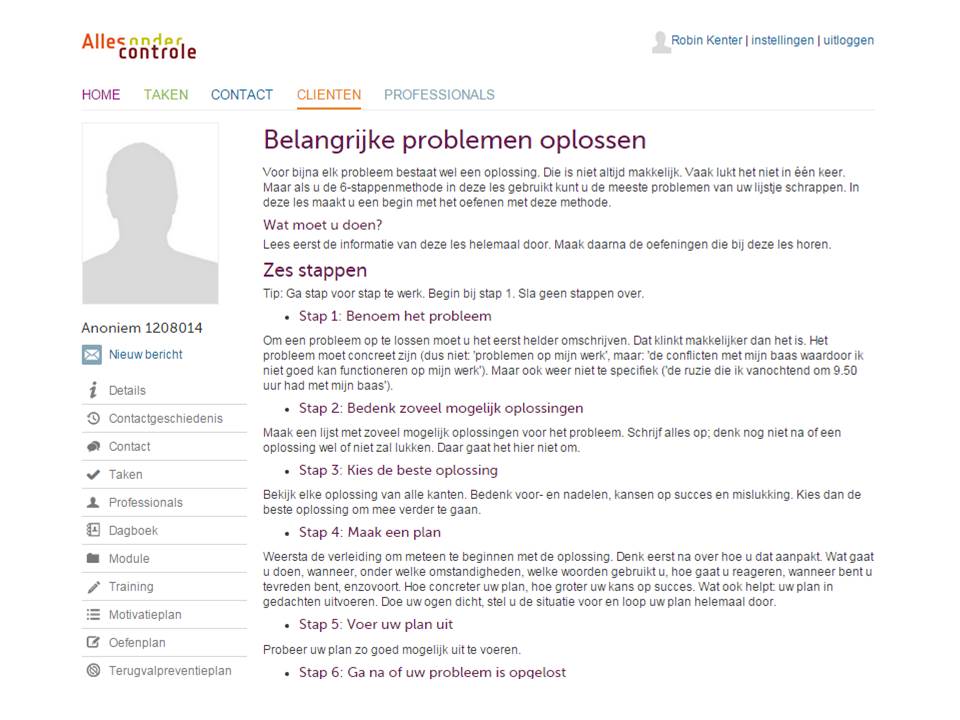

Supplement: Supplementary file 1 [file jmir_v18i3e80_app1.JPG]
